# P-1807. A Study on Clinical, Laboratory Parameters and Outcome Among Patients Infected with Different Dengue Serotypes in a Tertiary Care Hospital

**DOI:** 10.1093/ofid/ofaf695.1976

**Published:** 2026-01-11

**Authors:** Hardik Bharatbhai Patel, Bibhuti Saha, Soumendra Nath Haldar

**Affiliations:** SCHOOL OF TROPICAL MEDICINE, KOLKATA, West Bengal, India; SCHOOL OF TROPICAL MEDICINE, KOLKATA, West Bengal, India; SCHOOL OF TROPICAL MEDICINE, KOLKATA, West Bengal, India

## Abstract

**Background:**

This study aims to assess and compare the clinical presentations, laboratory parameters, radiological findings, and outcomes of adult patients infected with different dengue serotypes presenting to a tertiary care hospital in Kolkata, India, between July 2023 and April 2024

**Figure:**
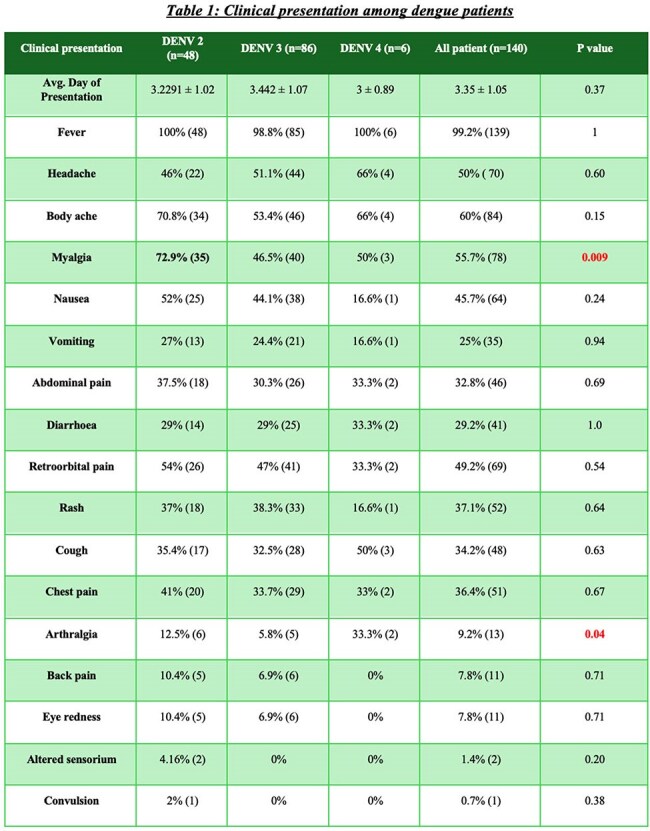
Clinical presentations among different Dengue serotypes Examination & Radiological findings among different Dengue serotypes

**Figure:**
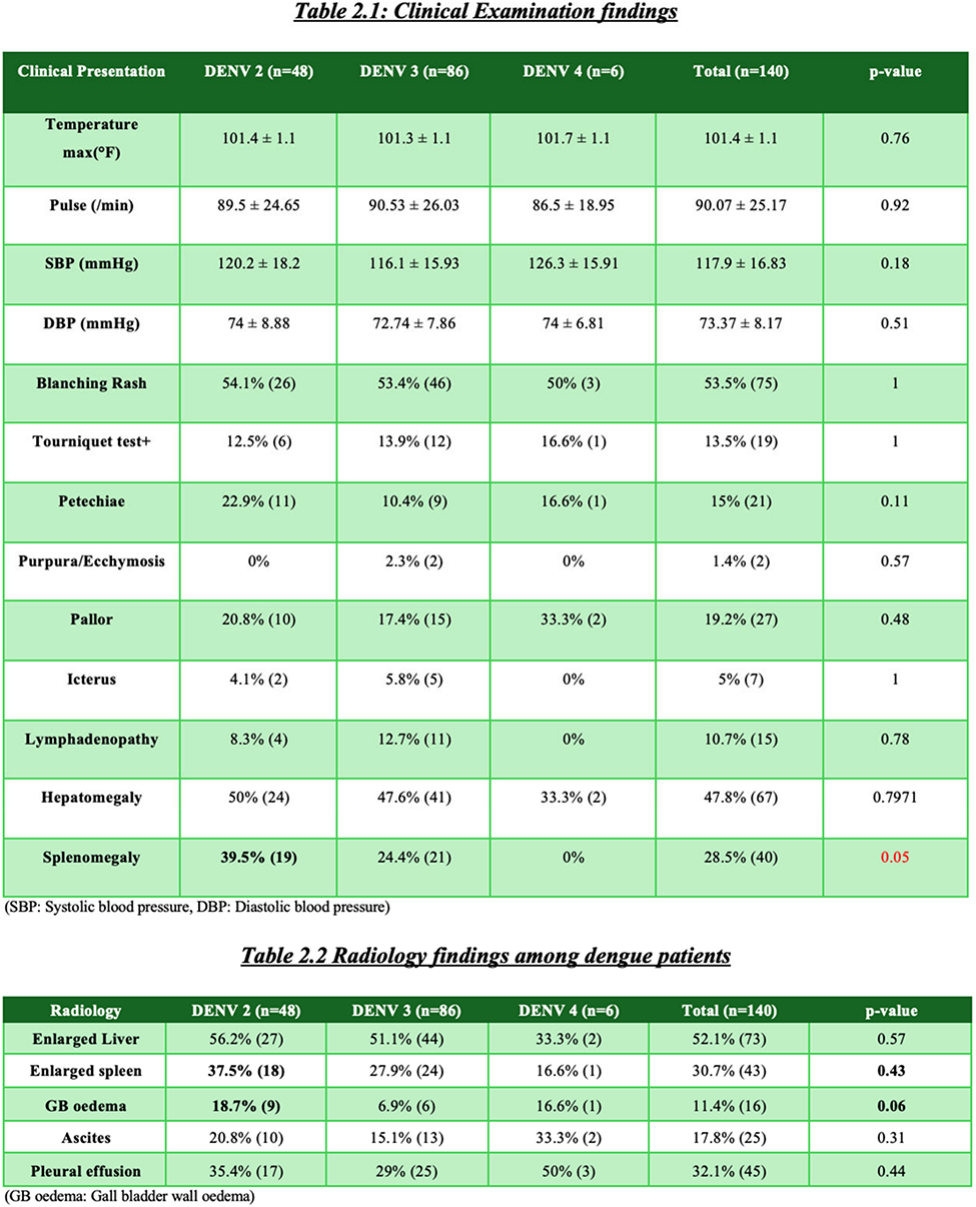

**Methods:**

A prospective observational study was done on 140 dengue-positive patients aged >18 years. Other infections were excluded. Clinical features, laboratory parameters, imaging, and outcomes were analyzed across serotypes using R software

**Figure:**
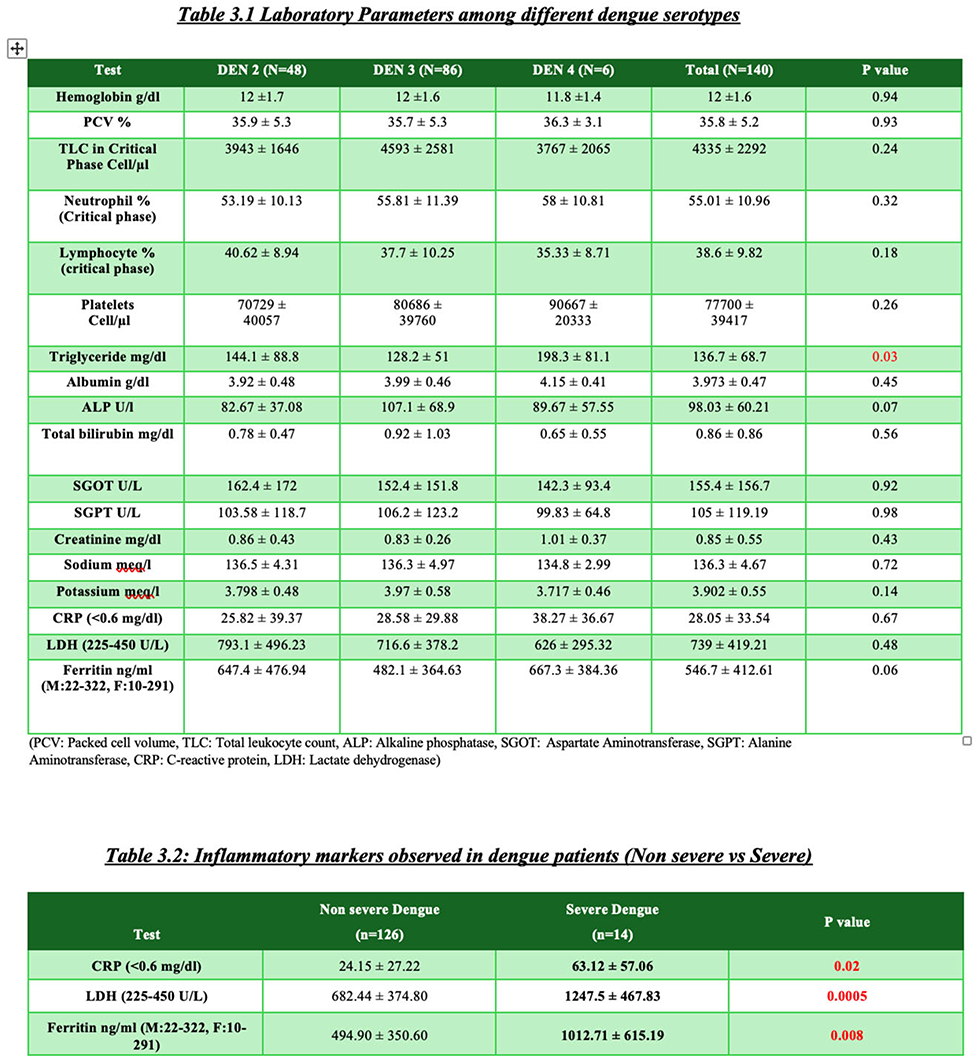
Laboratory parameters among different Dengue serotypes and Inflammatory markers non severe vs severe Dengue

**Figure:**
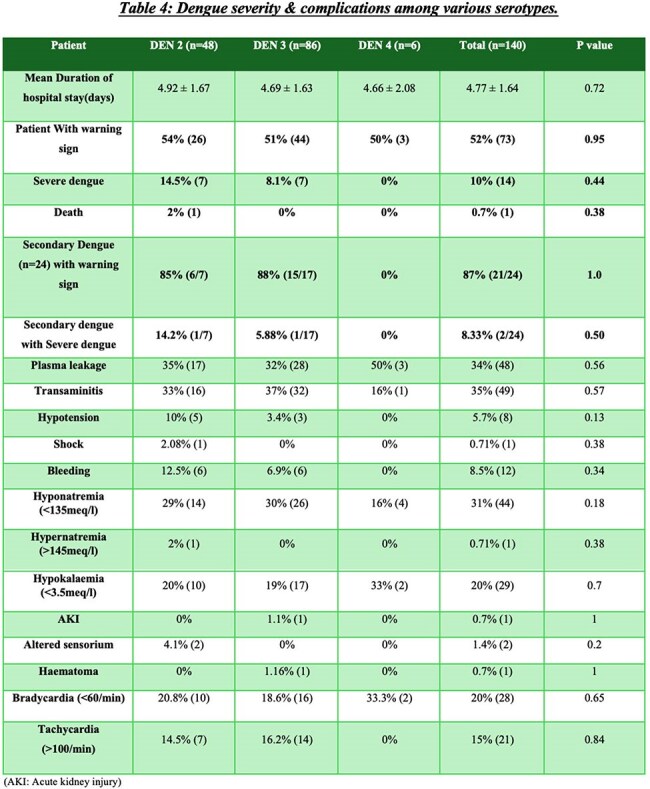
Dengue severity and complications among different Dengue serotypes

**Results:**

140 cases (DENV2:48, DENV3:86, DENV4:6) mean age 34.4±12 years; 55.5% male : 44.5% female. 77 patients needed admission. Hypertension(36%) commonest comorbidity. All comorbidities found no severity difference among three groups. Common symptoms fever (99%), body ache (60%), myalgia (55%), headache (50%), and nausea (45%). Myalgia was more in DENV2 (p=0.009), arthralgia in DENV4 (p=0.04). Hematological, liver function tests, renal function tests were similar across 3 groups, except for higher triglycerides (198 mg/dL) and CRP (38.2 mg/dL) in DENV4; ferritin was lowest in DENV3 (482 ng/mL). On imaging gall bladder wall edema (18%) and splenomegaly (37%) were more seen in DENV2. No significant difference in dengue with warning signs across groups. Severe dengue(n=14) occurred in 14% DENV2 and 8% DENV3, none in DENV4. Secondary dengue (n=24) showed more warning signs than primary (87% vs 44%, p=0.001). Complications like plasma leakage, shock, and bleeding were comparable across serotypes without significant difference. One death occurred in a DENV2 patient due to DSS

**Conclusion:**

DENV2 and DENV3 were predominant serotypes. DENV2 showed more systemic symptoms and notable radiological changes. Elevated inflammatory markers were linked to severity. No major difference in severity or outcome were found across serotypes. Study limitation was no DENV1 and few DENV4 cases. Other studies showed various serotype severity, this study adds to existing knowledge showing minimal outcome variation by serotype. Larger multicentric studies and serotype surveillance are needed to clarify serotype-related risks as dengue virus changes its genes & epidemiology. In my knowledge this is latest study (2023-2024) from India comparing dengue serotypes difference

**Disclosures:**

All Authors: No reported disclosures

